# High-affinity consensus binding of target RNAs by the STAR/GSG proteins GLD-1, STAR-2 and Quaking

**DOI:** 10.1186/1471-2199-11-48

**Published:** 2010-06-23

**Authors:** Andrew B Carmel, Joann Wu, Katrina A Lehmann-Blount, James R Williamson

**Affiliations:** 1 Department of Molecular Biology, The Scripps Research Institute, 10550 North Torrey Pines Road, MB-33. La Jolla, California, 92037, USA; 2 Department of Chemistry, The Scripps Research Institute, 10550 North Torrey Pines Road, MB-33. La Jolla, California, 92037, USA; 3 Skaggs Institute for Chemical Biology, The Scripps Research Institute, 10550 North Torrey Pines Road, MB-33. La Jolla, California, 92037, USA

## Abstract

**Background:**

STAR/GSG proteins regulate gene expression in metazoans by binding consensus sites in the 5' or 3' UTRs of target mRNA transcripts. Owing to the high degree of homology across the STAR domain, most STAR proteins recognize similar RNA consensus sequences. Previously, the consensus for a number of well-characterized STAR proteins was defined as a hexameric sequence, referred to as the SBE, for **S**TAR protein **b**inding **e**lement. *C. elegans *GLD-1 and mouse Quaking (Qk-1) are two representative STAR proteins that bind similar consensus hexamers, which differ only in the preferred nucleotide identities at certain positions. Earlier reports also identified partial consensus elements located upstream or downstream of a canonical consensus hexamer in target RNAs, although the relative contribution of these sequences to the overall binding energy remains less well understood. Additionally, a recently identified STAR protein called STAR-2 from *C. elegans *is thought to bind target RNA consensus sites similar to that of GLD-1 and Qk-1.

**Results:**

Here, a combination of fluorescence-polarization and gel mobility shift assays was used to demonstrate that STAR-2 binds to a similar RNA consensus as GLD-1 and Qk-1. These assays were also used to further delineate the contributions of each hexamer consensus nucleotide to high-affinity binding by GLD-1, Qk-1 and STAR-2 in a variety of RNA contexts. In addition, the effects of inserting additional full or partial consensus elements upstream or downstream of a canonical hexamer in target RNAs were also measured to better define the sequence elements and RNA architecture recognized by different STAR proteins.

**Conclusions:**

The results presented here indicate that a single hexameric consensus is sufficient for high-affinity RNA binding by STAR proteins, and that upstream or downstream partial consensus elements may alter binding affinities depending on the sequence and spacing. The general requirements determined for high-affinity RNA binding by STAR proteins will help facilitate the identification of novel regulatory targets *in vivo*.

## Background

Gene expression is regulated at the post-transcriptional level as a means of ensuring the proper localization and timing of developmental processes in eukaryotic organisms [1]. In a diverse set of pathways, a central feature of this post-transcriptional control involves specific binding to target RNA sequences by proteins belonging to the **S**ignal **T**ransduction and **A**ctivation of **R**NA/**G**RP33, **S**am68, **G**LD-1 (STAR/GSG) family. STAR protein-RNA interactions are important for translational silencing of genes necessary for germline fate in hermaphrodite *C. elegans *worms by the regulatory STAR protein GLD-1 [2,3], mRNA localization and subsequent development of central nervous system components in mice by Quaking (Qk-1) [4-6] and pre-mRNA binding by yeast BBP (**B**ranchpoint sequence **B**inding **P**rotein) and mammalian **s**plicing **f**actor 1 (SF-1) proteins as precursors for splicing into mature mRNA [7,8].

**Figure 1:**
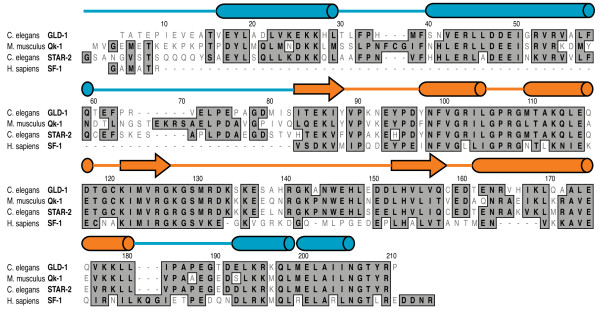
**STAR domain sequence alignment between *C. elegans *GLD-1 and STAR-2, mouse Qk-1 and human SF-1**. Positions with identical or similar amino acid residues are boxed in dark gray and differences are unboxed in white. Secondary structure elements above the sequence alignment are from the crystal structure of the GLD-1 Qua1 domain [15] (PDB ID 3K6T) and the solution structure of the SF-1 KH-Qua2 domains [11] (PDB ID 1K1G) with α-helices and β-strands represented by cylinders and arrows, respectively. Qua1 and Qua2 secondary structure domain boundaries are colored in blue and the central KH domain is colored in orange.

Members of the STAR/GSG protein family share a high degree of sequence similarity in the so-called STAR domain, defined by a central KH domain situated between two homologous domains to the mouse *Quaking *gene, called Qua1 and Qua2 (Figure 1) [9,10]. The KH and Qua2 domains provide an extended platform for RNA binding, as seen in the structure of the truncated SF-1 STAR domain in complex with single-stranded RNA, while Qua1 facilitates STAR homodimerization in the presence or absence of RNA [11-14]. Although an intact structure of a full STAR domain has yet to be determined, a recent crystal structure reveals that the GLD-1 Qua1 domain forms a helix-turn-helix motif which defines the homodimerization interface [15], while a solution structure of the free KH-Qua2 RNA binding region from the *Xenopus *Quaking protein provides additional information on the overall topology of this domain in the absence of a bound ligand [16].

In *C. elegans*, GLD-1 initiates the formation of a multi-protein repression complex that silences *tra-2 *translation by binding the 3' untranslated region (UTR) of *tra-2 *mRNA within 28-nt regulatory elements called TGEs (**T**RA-2 and **G**LI **E**lements) [2,17,18]. GLD-1 optimally recognizes a 5'-UACUCA-3' consensus sequence in the TGE plus an upstream dinucleotide contributes to the overall binding energy [12]. A comprehensive mutational analysis further identified other permissible nucleotide sequences that GLD-1 binds with slightly lower affinity [12], and numerous potential targets for GLD-1 mediated regulation have since been identified by the presence of a relaxed consensus hexamer with the sequence 5'-(U > G > C/A)A(C > A)U(C/A)A-3' in the 5' or 3' UTRs of these mRNAs.

A similar hexameric target was also identified for the mouse Quaking protein (Qk-1) having the sequence 5'-NA(A > C)U(A>>C)A-3' [19]. *In vivo*, Qk-1 facilitates the proper sub-cellular localization and expression of **m**yelin **b**asic **p**rotein (MBP) in glial cells as a direct result of interactions with consensus binding sites in MBP mRNA [4,6]. Qk-1 binds substantially tighter to a 5'-UAAUAA-3' consensus, different than GLD-1 in the strong preference for adenosine at both the third and fifth positions [19]. Subsequent reports expanded the Qk-1 binding site to include additional upstream or downstream partial consensus sequences, although the role of these elements in Qk-1 binding remains less well understood [20,21].

SF-1 is another well-characterized STAR protein which recognizes a 5'-UACUAAC-3' consensus in the **b**ranch **p**oint **s**equence (BPS) RNA that is identical within the sequence parameters delineated for GLD-1 and Qk-1 [7]. Additionally, Sam68 and the Sam68-like proteins SLM-1 and SLM-2 recognize bipartite RNA elements that resemble the canonical consensus sequences bound tightly by GLD-1 and Qk-1 [21].

Based largely on sequence homology, novel STAR proteins have been identified whose functions and biological roles remain largely uncharacterized. One such protein called STAR-2 (also called ASD-2 for **a**lternative **s**plicing **d**efective-2) is thought to be a functional GLD-1 homolog expressed in *C. elegans *somatic tissues. In a recent report, STAR-2 was shown to play an important role in *C. elegans *development by facilitating alternative splicing patterns in *let-2 *mRNA and regulating expression of *let-2 *[22]. Given the 67% sequence identity with GLD-1 in the STAR domain (Figure 1), it seemed likely that STAR-2 would bind similar RNA target sequences as GLD-1 and so may perform an analogous regulatory role. To test this, a combination of gel mobility shifts and fluorescence-polarization was used to assess whether STAR-2 binds TGE RNA with an affinity and specificity comparable to GLD-1. Interestingly, STAR-2 binds a similar consensus as GLD-1, but more closely resembles Qk-1 in the strong preference for adenosine at the third and fifth consensus positions. The relative competitive efficiency of mutant 12-mer RNA sequences containing a single hexamer site was then tested against STAR-2 bound to a modified TGE RNA in the more appropriate consensus site background.

Gel mobility shifts and fluorescence-polarization were then used to further detail the contributions of individual hexamer consensus positions for high-affinity binding by GLD-1 and Qk-1. Because wild-type TGE does not contain the consensus sequence most preferred by Qk-1, a modified TGE RNA with the tightest binding Qk-1 consensus was used as a probe in a competition fluorescence-polarization assay to better define the sequence requirements for high-affinity RNA interactions. The role of full and partial consensus elements situated upstream or downstream of a canonical hexamer was also examined in a variety of RNA backgrounds. These results indicate that a single consensus hexamer is sufficient for tight binding by STAR proteins and additional upstream or downstream consensus elements may enhance binding depending on the sequence and positioning.

## Results

### STAR-2 binds TGE RNA with high affinity

**Figure 2:**
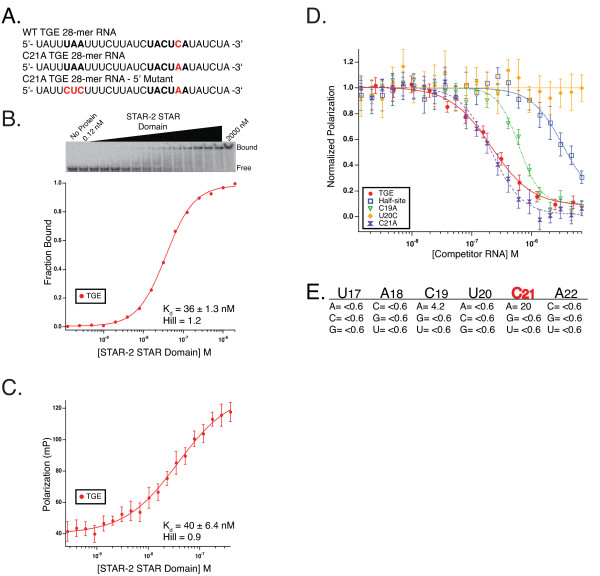
**STAR-2 binding to TGE RNA and determination of STAR-2 binding specificity**. **A**. Wild-type TGE RNA compared to sequences for C21A TGE and TGE with changes in the upstream 5'-UAA element. **B**. Electrophoretic gel mobility shift titration of the STAR-2 STAR Domain into radiolabeled TGE RNA with STAR-2 concentration and bound complex denoted. The equilibrium dissociation constant and Hill values shown were calculated from the plot of fraction bound as a function of STAR-2 concentration. **C**. STAR-2 STAR Domain binding to fluorescein-labeled TGE RNA measured by fluorescence-polarization. K_d _and Hill values shown were determined from the fit of the polarization versus concentration of STAR-2. Error bars represent the standard deviation of 10 consecutive readings from the same plate. **D**. Plot of the normalized polarization against concentration of mutant 12-mer competitor RNAs. Traces display the competitive efficiency of three point mutants, wild-type TGE half-site and self-competition with the 28-nt TGE. **E**. Table of relative IC_50 _values for the mutant 12-mer RNA sequences compared to wild-type TGE half-site.

STAR-2 shares a high degree of sequence identity with GLD-1 in the STAR domain region (Figure 1) and sequence similarity suggests that STAR-2 may represent the somatic homolog of the germline GLD-1 protein in *C. elegans*. To address this, the K_d _between the STAR-2 STAR domain (STAR-2-STAR) and wild-type TGE RNA (Figure 2A) was determined both by gel mobility shift (Figure 2B) and fluorescence-polarization (Figure 2C). STAR-2-STAR binds TGE RNA with 36 ± 1.3 nM and 40 ± 6.4 nM affinity by these methods, respectively. These values are similar to the previously reported K_d _for GLD-1 using gel shift (11.4 ± 2 nM) [12] and are within error to a measured K_d _of 32 ± 3 nM for GLD-1 by fluorescence-polarization (data not shown), suggesting that STAR-2 binds TGE RNA in a manner comparable to that of GLD-1. While STAR-2 and GLD-1 bind TGE RNA with similar affinities and the sequence identity between these two proteins suggested a similar binding site, the sequence requirements for binding still needed to be determined.

### Determination of STAR-2 binding specificity

Competition fluorescence-polarization was used to probe the nucleotide sequence identity of the consensus recognized by STAR-2 with the same 12-mer TGE RNA library used previously for GLD-1 [12]. Each 12-mer RNA, containing a single nucleotide substitution in the wild-type 5'-UACUCA-3' consensus, was tested for it's ability to interfere with a STAR-2-STAR/TGE complex. Of the 18 RNAs, only two point mutations in the 12-mer library, C19A and C21A, bind as tight or tighter than wild-type TGE half-site, 4-fold and 20-fold for each substitution (relative IC_50 _half site/C19A = 4.2; relative IC_50 _half-site/C21A = 20), respectively (Figure 2D, 2E).

Interestingly, because GLD-1 binds only slightly tighter to the 12-mer C21A variant (data not shown), the 20-fold preference by STAR-2 for an adenosine at the fifth position more closely resembles Qk-1, which binds the C21A half-site 41-fold tighter by this method [19]. Similarly, wild-type 12-mer competes relatively poorly for binding to STAR-2-STAR, 7-fold weaker than the 28-nt TGE self-competition (relative IC_50 _TGE/wild-type half-site = 0.13) while the C21A 12-mer binds nearly 3 fold better than the full-length TGE (relative IC_50 _TGE/C21A 12-mer = 2.7) just as with Qk-1. Furthermore, STAR-2-STAR binds the C19A 12-mer 4-fold tighter than the wild-type half-site, similar to that seen for this RNA by Qk-1, while GLD-1 has a slight preference for cytidine at this position over adenosine. STAR-2 binds most tightly to RNA sequences containing the consensus 5'-UA(A>C)U(A>>C)A-3', identical to the high-affinity consensus binding site for Qk-1, but still within the sequence parameters of the relaxed consensus identified for GLD-1.

**Figure 3:**
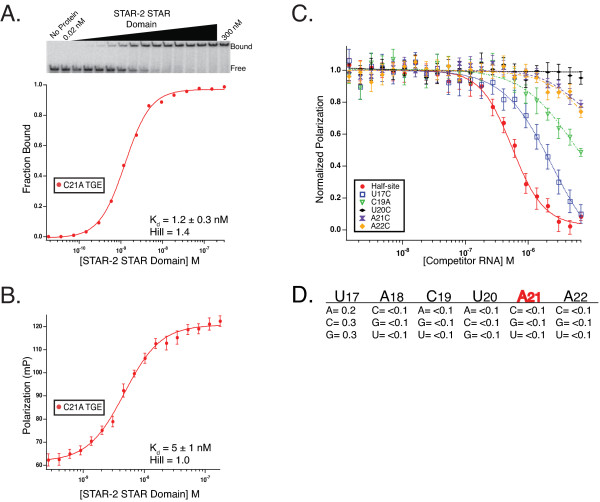
**STAR-2 RNA binding affinity in C21A consensus background**. **A**. Direct titration of the STAR-2 STAR Domain into radiolabeled C21A TGE RNA by EMSA with K_d _and Hill coefficient values displayed. **B**. Fluorescence-polarization used to measure binding of the STAR-2 STAR Domain to C21A TGE RNA. **C**. Competition fluorescence-polarization for 12-mer point-mutants in the C21A background. The 28-nt C21A TGE RNA was used as a probe with traces for A21 half-site RNA and four mutants shown. **D**. Relative IC_50 _values for mutant 12-mer RNAs compared to A21 half-site RNA in the A21 consensus background.

Next, both EMSA and fluorescence-polarization were used to measure the K_d _between STAR-2-STAR and 28-nt TGE RNA containing the C21A substitution in the consensus (Figures 2A, 3A and 3B). STAR-2-STAR binds C21A TGE with 1.2 ± 0.3 nM and 5 ± 1 nM affinity by each method, respectively, which is essentially identical to the affinity of Qk-1-STAR for this RNA (see Table 1). For comparison, GLD-1-STAR binds C21A TGE RNA only slightly tighter than wild-type TGE by fluorescence-polarization (data not shown), which agrees well with the 12-mer competition binding results indicating that GLD-1 is relatively indifferent to either cytidine or adenosine at that position.

**Table 1:** Summary of Qk-1 binding to 28-mer RNAs

RNA	K_d _(nM) EMSA	K_d _(nM) FPA
WT TGE	49 ± 2	63 ± 4
C21A TGE	1.2 ± 0.2	13 ± 2
C21A 5' Mutant	7.1 ± 1	----

To more effectively define the contribution of each nucleotide for high-affinity STAR-2 binding, a competitor library of 18 oligonucleotides, synthesized in a background containing the 5'-UACUAA-3' consensus sequence optimal for STAR-2, was used for competition fluorescence-polarization (Figure 3C, 3D). In this assay, no single point mutation competes effectively with the 28-nt C21A TGE for binding STAR-2-STAR except for the A21 half-site 12-mer. Neither the wild-type TGE half-site with the A to C "reversion" mutation or C19A was found to bind with high-affinity when the bound probe contains adenosine at the fifth position. In addition, STAR-2 prefers uracil at the first position, but all other nucleotide substitutions compete within 5-fold affinity of the A21 half-site.

### Qk-1 and STAR-2 bind identical hexanucleotide consensus sequences

**Figure 4:**
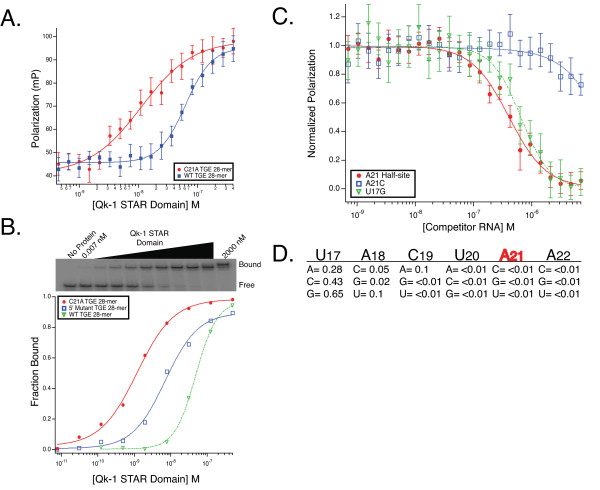
**Qk-1-STAR binding to TGE RNA and consensus site mutants by fluorescence-polarization and EMSA**. **A**. Plot showing polarization as a function of protein concentration for the direct titration of Qk-1 STAR Domain into fluorescein-labeled TGE RNA or the C21A TGE variant. **B**. Comparative plot of Qk-1-STAR titrations into radiolabeled TGE RNA, C21A TGE RNA or the 5' mutant TGE 28-mer. The gel mobility shift of Qk-1-STAR into C21A TGE RNA is shown with the bound and free species and the concentration of Qk-1-STAR through the titration indicated. **C**. Competition binding with the normalized polarization plotted against the concentration of unlabeled competitor 12-mer RNA. Shown are traces for the A21 12-mer half-site RNA and two mutant 12-mer sequences where each was titrated into a constant concentration of Qk-1-STAR in complex with C21A TGE RNA. **D**. Relative IC_50 _values for each of the 12-mer mutants compared to the A21 half-site RNA.

Qk-1 recognizes sites in the UTRs of target mRNA transcripts, binding tightest to consensus hexamer sequences containing adenosine rather than cytidine at the fifth position. Here, Qk-1-STAR binding affinity for wild-type TGE RNA (5'-UACUCA-3') or the C21A variant (5'-UACUAA-3') (Figure 2A) was compared by direct titration using both fluorescence-polarization (Figure 4A) and gel mobility shift (Figure 4B). Qk-1 binds much tighter to 28-mer RNAs containing a 5'-UACUAA-3' hexamer, which is underscored by the five-fold tighter binding to C21A TGE RNA than wild-type seen by fluorescence-polarization and nearly 41-fold tighter binding as measured by EMSA (K_d _values are summarized in Table 1).

Competition fluorescence-polarization was then used to measure the ability of each mutant 12-mer RNA, representing all 18 point mutations in the preferred 5'-UACUAA-3' consensus, to compete against the 28-nt C21A TGE variant in complex with Qk-1-STAR. In this assay, Qk-1-STAR has a slight preference for uracil at the first position (U17) but will tolerate any of the other three nucleotides nearly as well (Figure 4C, 4D). Interestingly, no other single mutant 12-mer competes measurably with the C21A TGE RNA, including A21C (wild-type TGE half-site) and C19A, the only effective competitor being the A21 half-site 12-mer RNA (5'-AUC**UACUAA**UAU-3'; K_i_^app ^= 46 nM). These results highlight that the strongest determinant for high-affinity binding is a hexameric sequence with adenosine at the fifth position, as even C19A does not confer any additional binding specificity in this context.

### Upstream elements contribute to high-affinity binding by Qk-1

In addition to a hexanucleotide consensus, GLD-1 recognizes an upstream 5'-UA sequence in the TGE (Figure 2A) [12]. Partial consensus elements situated upstream or downstream of a canonical hexamer in RNA sequences bound tightly by Qk-1 have also been noted previously, although the quantitative importance of the upstream 5'-UA for Qk-1 binding to TGE RNA has not been determined [19-21]. A modified TGE RNA in the 5'-UACUAA-3' consensus background was created by substituting 5'-CUC for the upstream partial consensus 5'-UAA element (Figure 2A). This RNA was used in a gel mobility shift assay to measure the binding affinity to Qk-1-STAR by direct titration (Figure 4B). Qk-1-STAR binds the 5' mutant TGE RNA with roughly 6-fold reduced affinity compared to the C21A TGE (7.1 ± 1 vs. 1.2 ± 0.2 nM; Table 1) but still considerably tighter than to wild-type TGE RNA. This result is consistent with the modest contribution of this upstream element for GLD-1 binding to TGE RNA and further indicates that a 5'-UA(A/C)UAA-3' hexanucleotide consensus is the most important feature for high-affinity binding by Qk-1.

### Alternative mutations in MBP consensus restore high-affinity binding by Qk-1

Previously, Ryder et al. described the GLD-1 and Qk-1 high-affinity binding sites as consensus hexamer sequences [12,19]. In a subsequent report based on *in vitro *selection, the Qk-1 binding site was expanded to include a "half-site" positioned upstream or downstream of a consensus hexamer that further defined the STAR binding consensus as a bipartite element [20]. In that report, nucleotides surrounding the hexanucleotide consensus were viewed as crucial for Qk-1 recognition because binding was virtually abolished when either the core hexamer or "half-site" was mutated in one of the high-affinity binding sites found in myelin basic protein (MBP) mRNA. This led the authors to conclude that both the core and "half site" must be critical for high-affinity binding if mutations in either one rendered Qk-1 unable to bind. However, we propose as an alternative that these particular core and "half site" mutations caused unforeseen secondary structure changes in MBP RNA that prevented Qk-1 from binding by potentially masking the high-affinity hexameric consensus site.

**Figure 5:**
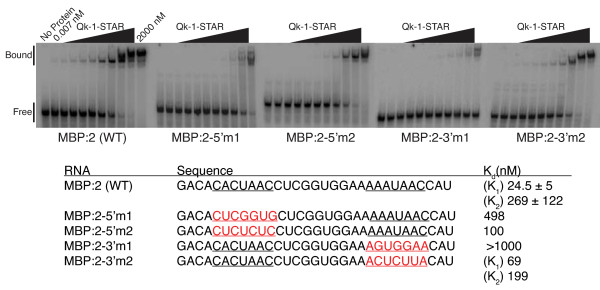
**EMSA analysis of Qk-1-STAR binding to RNA fragments from the 3' UTR of MBP RNA**. The wild-type sequence (MBP:2 (WT)) contains both 5' and 3' consensus sequences, underlined in black. Nucleotide changes in either the 5' (MBP:2-5'm1 or m2) or 3' (MBP:2-3'm1 or m2) consensus sites are displayed in the mutant sequences by underlining in red text. In each case, Qk-1-STAR was titrated into a reaction mixture with radiolabeled MBP RNA. The bound complex and free RNA bands are indicated with binding dissociation constants listed for each RNA. K_d _values were calculated independently for each distinct shift (K_1 _and K_2_) for WT and 3'm2 RNAs.

To address this issue, the consensus core and "half-site" elements were both altered using more conservative mutations, and these MBP RNA variant sequences were tested for binding to Qk-1-STAR in a gel mobility shift assay (Figure 5). The wild-type sequence represents 31-nt surrounding the second of five consensus binding sites previously identified in the 3' UTR of MBP RNA [19,20]. This RNA contains a 5' "core" consensus with sequence 5'-CACUAA-3' and a downstream 3' "half-site" with sequence 5'-UAAC-3', as previously described [20]. Qk-1-STAR binds this RNA tightly and the presence of a second distinct shifted complex in the gel indicates that an additional Qk-1-STAR dimer may bind as well (Figure 5). The first and second binding events have measured affinities of 24.5 ± 5 nM and 269 ± 122 nM, respectively, suggesting that Qk-1 binds one site preferentially. In effect, either could be considered the preferred consensus site since a second high-affinity hexameric consensus element with sequence 5'-AAAUAA-3' is seen by including nucleotides just upstream of the 3' "half-site". This second shifted complex also appears in the previously published gel for this RNA, although only a single binding constant of 115 nM was reported [20].

**Figure 6:**
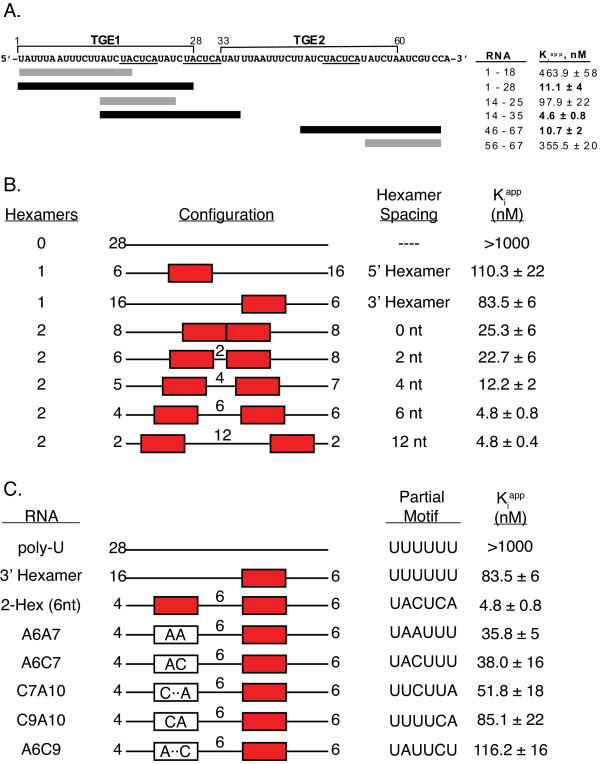
**TGE deletions, dual hexamer spacing requirements and upstream partial consensus elements define GLD-1 dimer binding**. **A**. The two TGE region of the *tra-2 *mRNA 3' UTR is shown with the 28-nt TGE boundaries delineated and the three canonical 5'-UACUCA-3' hexamers underlined. Bars beneath the sequence correspond to the regions tested and are colored according to the calculated inhibition constant. RNAs with a K_i_^app ^≤ 15 nM are represented by black bars and RNAs that compete with the native TGE RNA less effectively are represented by grey bars. Each RNA is listed by the position in the sequence with the corresponding K_i_^app ^value. **B**. The spacing requirements for canonical hexamer placement in a 28-nt poly-uridine background. Consensus 5'-UACUCA-3' hexamers are displayed as red boxes. The relative position of each hexamer is shown with the numbers indicating nucleotide spacing between hexamers and hexamer distance from either the 5' or 3' end. **C**. High-affinity GLD-1 binding and the effects of various dinucleotide insertions upstream of a canonical hexamer in a poly-uridine background. Each RNA contains a single 5'-UACUCA-3' hexamer (red boxes) positioned 6 nucleotides from the upstream partial motif, with the numbering referring to the hexamer boundary distances from the 5' and 3' ends. The partial motif sequences are given and are shown schematically within the white boxes. Uridine residues are indicated by dots. The effect of added A or C residues was evaluated in comparison to 5'-UUUUUU-3'.

As previously noted, Qk-1-STAR does not bind MBP RNA when the 5' consensus site is changed from 5'-CACUAAC-3' to 5'-C**U**C**GGUG**-3' or when the 3' "half-site" is altered from 5'-AAAUAAC-3' to 5-A**GUGG**A**A**-3' (Figure 5). A direct titration gel shift was used to monitor the ability of Qk-1-STAR to bind MBP RNA with these mutations. Qk-1-STAR binds the 5' mutant (labeled MBP:2-5'm1 in Figure 6) relatively weakly (K_d _= 498 nM) in this assay while virtually no binding was observed to the 3' mutant (MBP:2-3'm1) in accordance with previous results [20].

These results seemingly contradict the idea of a hexanucleotide consensus as the primary specificity determinant for Qk-1 binding. Both MBP:2-5'm1 and MBP:2-3'm1 have valid hexanucleotide elements, but it seemed possible that introduction of multiple G residues in an A/U rich RNA sequence might result in the formation of secondary structures that inadvertently sequester the hexanucleotide element. Indeed, RNA secondary structure predictions using mfold [23,24] suggest that both MBP:2-5'm1 and MBP:2-3'm1 contain highly stable structured regions that mask the high-affinity binding sites in these RNAs (data not shown). Furthermore, tight binding to these sequences is restored when more conservative mutations are made in either the 5' or 3' sites. Replacing the 5' half-site with the sequence 5'-C**U**CU**CU**C-3' (MBP:2-5'm2) results in nearly 5-fold tighter binding (K_d _= 100 nM) when compared to MBP:2-5'm1. Similarly, Qk-1-STAR binds tightly to MBP:2-3'm2 and the two distinct complexes seen in the gel have measured K_d _values of 69 nM and 199 nM for the first and second shifts, respectively. These values are nearly identical to that for wild-type MBP:2 RNA and neither of these mutant RNAs contain the stable secondary structures in mfold predictions as seen for MBP:2-5'm1 or MBP:2-3'm1. In addition, by simply adding multiple guanosine residues to the 5' end of the TGE in order to facilitate *in vitro *transcription using T7 polymerase, GLD-1 binding was essentially abolished, presumably due to formation of stable secondary structures in the RNA (data not shown). Taken together, these results suggest that MBP:2 RNA contains two independent consensus binding sites and that only one is necessary for Qk-1 to bind with high-affinity. In addition, Qk-1 is indifferent to the positioning of the consensus site as it bound the full-length MBP RNA with roughly the same affinity when either the 5' or 3' consensus sites were mutated.

### GLD-1-STAR binds tighter to RNA with two consensus hexamers

One poorly understood aspect of this system is the mode that homodimeric STAR proteins employ when binding target RNA sites, since structural models are based largely on the solution structure of monomeric human SF-1 in complex with Branch Point Sequence (BPS) RNA [11]. SF-1 lacks the Qua1 region and does not dimerize, making the structure only illustrative as a model for RNA binding by a single STAR monomer [11]. Furthermore GLD-1, Qk-1 and STAR-2 bind much tighter to their target RNA sequences than SF-1 does to its consensus RNA *in vitro*, and this difference may be due in large part to the extra binding energy attained from recognition of additional RNA elements by the second protomer.

Competition fluorescence-polarization was used to address this issue by measuring the affinity of GLD-1-STAR for various TGE RNAs, truncated so as to contain zero, one or two consensus hexamers (Figure 6A). The *tra-2 *mRNA 3'-UTR contains two 28-nt TGE repeats each with a single consensus hexamer, plus an additional hexamer encompassing part of the conserved 5'-CUCA-3' in the linker separating the two TGEs. Neither of the two TGE truncations lacking a consensus element (1-18 and 56-67) compete well with the fluorescently-labeled 28-nt TGE for GLD-1-STAR binding. When compared to self-competition with the 28-nt TGE, 1-18 and 56-67 bind 42 and 32-fold weaker, respectively, underscoring the necessity of a hexamer consensus for high-affinity binding.

GLD-1-STAR binds the TGE half-site RNA (labeled 14-25) containing one consensus hexamer roughly 9-fold weaker than the TGE. This is consistent with the previously measured values for this RNA by both Fp and EMSA [12,19]. Weaker binding to the half-site is likely due to the lack of any additional recognition elements in the shorter 12-nt RNA since tight binding is restored with the longer 46-67 RNA, which has both a consensus hexamer and a downstream UAA element. GLD-1-STAR binds this RNA nearly ten-fold tighter compared to the half-site and binds as tightly as to the full 28-nt TGE (10.7 ± 2 nM versus 11.1 ± 4 nM). Among these sequences, GLD-1-STAR binds the tightest to the 14-35 RNA containing two consensus hexamers (K_i_^app ^= 4.6 ± 0.8 nM), binding this RNA nearly 2.5-fold tighter than the TGE 28-mer.

### Consensus site spacing is acceptable over a broad range

As a homodimer, both protomers should be equally competent for binding a consensus element in the context of a dual hexamer RNA. However, it remained unclear whether enhanced binding occurs only when the consensus sites are spaced within a certain permissible range. To this end, a library of RNA constructs was developed that placed either one or two canonical hexamers at different locations within a poly-uridine background and were tested for binding to GLD-1-STAR by competition fluorescence-polarization (Figure 6B). The 28-nt TGE sequence is 54% uridine (15 of 28 bases) and a 28-mer RNA consisting entirely of uridine does not compete for GLD-1-STAR binding (Figure 6B).

Addition of one 5'-UACUCA-3' hexamer in the poly-U background at either the 5' or 3' end resulted in a K_i_^app ^of 110.3 ± 22 nM or 83.5 ± 6 nM, respectively, which is similar to that observed for the half-site 12-mer (14-25) containing a single hexamer and also consistent with the affinity of GLD-1-STAR for TGE RNA with mutations in the upstream UA element [12]. Addition of a second hexamer restores tighter binding to a similar level as that of the 28-nt TGE when there is a 4-nt spacing between hexamers (4-nt, K_i_^app ^= 12.2 ± 2 nM). Binding affinity is 2-fold weaker than TGE RNA when the hexamers are directly adjacent to each other (0-nt, K_i_^app ^= 25.3 ± 6 nM) or with a 2-nt spacer (2-nt, K_i_^app ^= 22.7 ± 6 nM). A spacing of six to twelve nucleotides between hexamers was optimal for tight binding with K_i_^app ^values of 4.8 ± 0.8 nM and 4.8 ± 0.4 nM for a 6-nt and 12-nt spacing, respectively.

In the same 28-nt poly-U RNA background discussed above, various dinucleotide sequences were then inserted upstream of a single consensus hexamer and the contributions of these smaller sequence motifs to GLD-1 binding affinity were evaluated by competition fluorescence-polarization (Figure 6C). None of the partial motifs recapitulated the effect of adding a second consensus hexamer, although three combinations of dinucleotide substitutions reflect a portion of a relaxed consensus element. Adding an upstream AA or AC (A6A7, 5'-U**AA**UUU-3'; A6C7, 5'-U**AC**UUU-3') was the most preferred, with a K_i_^app ^of 35.8 ± 5 nM and 38.0 ± 16 nM, respectively, but both are still nearly 8-fold reduced compared to the addition of a consensus hexamer (K_i_^app ^= 4.8 ± 0.8 nM). Interestingly, RNA with the C9A10 substitution (5'-UUUU**CA**-3') does not bind tighter than 5'-UUUUUU-3' (K_i_^app ^= 85.1 ± 22 nM versus K_i_^app ^= 83.5 ± 6 nM) even though this substitution places half of a 5'-UACUCA-3' consensus upstream of the full hexamer. Of the other sequences tried, C7A10 (5'-UU**C**UU**A**-3') and A6C9 (5'-U**A**UU**C**U-3') compete with a K_i_^app ^of 51.8 ± 18 and 116.2 ± 16 nM, respectively, indicating that these sequences do not enhance GLD-1 dimer binding when only a single consensus element is present.

## Discussion and Conclusions

STAR/GSG proteins regulate the expression of developmental genes in eukaryotes by binding specific sites in target mRNA transcripts. In *C. elegans *worms, GLD-1 binds to TGE RNA and regulates *tra-2 *translation, while in mice, Qk-1 binds MBP RNA and ensures the proper sub-cellular localization and expression of myelin. Both GLD-1 and Qk-1 bind tightly to a similar hexameric consensus element within their respective RNA targets but require different sequences for optimal binding (summarized in Table 2). Although there remains no clear explanation as to what is responsible for the distinct binding specificities of GLD-1 and Qk-1, this difference likely plays a role in the regulatory activity of each protein by preferred RNA target site selection *in vivo*.

**Table 2:** Optimal RNA hexamer consensus sequences for high-affinity STAR protein binding

STAR protein	Hexamer consensus
GLD-1	5'-(U > G > C/A)A(C > A)U(C/A)A-3'
Qk-1	5'-NA(A>C)U(A>>C)A-3'
STAR-2	5'-UA(A>C)U(A>>C)A-3'

This has been useful for identifying binding sites in potential RNA regulatory targets and for helping us to initially characterize the RNA binding activity of newly recognized STAR proteins. For example, STAR-2 was initially identified as a potential GLD-1 homolog in *C. elegans *somatic tissues. In this report, it was established that STAR-2 and GLD-1 bind similar hexameric consensus sequences (Table 2) and so may regulate gene expression in an analogous fashion. Although STAR-2 binding is tightest to a consensus hexamer that more closely resembles the sequence preferred by Qk-1 rather than GLD-1, this is consistent with the model that most STAR proteins recognize similar specificity determinants in the 5' or 3' UTR regions of target RNAs.

Although the consensus sequence requirements vary for each individual STAR protein, all of those studied in detail necessitate a hexameric consensus element at a minimum in order to bind RNA with high-affinity. However, while not an absolute requirement for tight RNA binding, upstream or downstream partial consensus elements play an integral role in high-affinity STAR binding that remains somewhat less understood. For instance, mutations in partial consensus sequences found in TGE and MBP RNA have an adverse effect on both GLD-1 and Qk-1 binding, respectively [12]. However, these results further validate the idea that only one hexameric consensus site is absolutely necessary and that upstream or downstream partial consensus elements play a secondary, but not essential role for high-affinity binding by STAR proteins.

**Figure 7:**
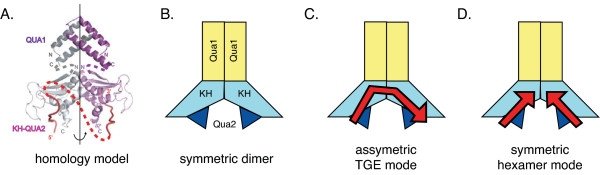
**Possible high-affinity RNA binding modes by STAR protein dimers**. **A**. Model for STAR protein dimerization using the monomeric structure of SF-1 bound to RNA aligned with the three-dimensional structure of the GLD-1 Qua1 dimer interface. The dashed red line represents the potential orientation of a contiguous stretch of single-stranded RNA connecting the two hexamer binding sites. **B**. Qua1-mediated STAR dimers likely present identical RNA binding surfaces by both protomers. The STAR domains are represented by polygons in yellow, light blue and dark blue corresponding to Qua1, KH and Qua2, respectively. **C**. In the case of GLD-1 bound to TGE RNA, the asymmetric complex would have one protomer binding the canonical hexamer in the usual mode while the other would recognize the upstream element in a distinct manner. **D**. Symmetric hexamer mode in the case of a 2:1 binding stoichiometry, as with a 12-mer RNA containing a single hexamer. In this case, each monomer binds RNA, represented here by red arrows, in an identical fashion.

One possible explanation for this suggests that partial consensus sequences may provide a binding platform for the second protomer of a STAR homodimer in a bound complex with RNA. In this case, the RNA has two unequal binding sites such that one STAR protomer binds the canonical consensus hexamer in a similar manner as that seen in the solution structure of SF-1 bound to RNA (Figure 7A) while the other protomer would bind the second partial consensus site in a novel fashion. The model showing the Qua1-mediated dimer interface was based on the crystal structure of the GLD-1 Qua1 domain [15] with the KH domains modeled after the orientation seen in the Nova KH domain crystal structure [25-27]. The dashed line in Figure 7A shows the approximate path of the contiguous RNA through the KH-Qua2 region, as would be the case for the TGE. A symmetric STAR homodimer shown schematically in Figure 7B presents two equal RNA binding surfaces and would necessarily bind TGE RNA asymmetrically as in Figure 7C. In this case, mutations in the upstream partial consensus that may render the second protomer unable to bind result in a weaker STAR protein-RNA complex, consistent with that seen for both GLD-1 [12] and Qk-1.

Most of the competition experiments described here involved assessing binding affinities using 12-mer RNAs containing only a single hexamer. It is possible based on this model, in which each protomer is equally competent for binding a consensus hexamer, that a STAR dimer may simultaneously bind two RNA 12-mers with each protomer recognizing a consensus hexamer in a symmetric fashion (Figure 7D). This has been termed the symmetric hexamer binding model and it may also describe the mode of binding to a dual hexamer RNA, such as that employed by GLD-1. As learned in this report, GLD-1 binds tightly to poly-U RNA containing two consensus hexamer sequences and the permissible spacing between hexamers varies over a fairly wide range. Symmetric binding is possible in this case only if the intervening RNA sequence is of sufficient length to properly orient the two consensus binding sites. Since GLD-1 binds the tightest to RNA with two consensus hexamers spaced between 6 and 12-nt apart, it is possible in this context for the RNA to be oriented such that each protomer interacts with a consensus hexamer in an identical manner. These binding consensus studies should prove useful for defining potential targets for the individual STAR proteins.

Other possible models that may explain high-affinity binding must take into consideration the likely orientation of the STAR protomers and the positioning of RNA elements that may contribute differentially to the binding energy of the complex. This highlights the difficulty in describing the interaction between STAR dimers and target RNA sequences without the benefit of a high-resolution structure. Hopefully, efforts currently underway to describe the structure of a full STAR domain will provide more insight on the mode of high-affinity RNA binding by STAR proteins in general.

## Methods

### Protein expression and purification

The STAR domains from GLD-1 and Qk-1 were overexpressed in *E. coli *as maltose binding protein (MBP) fusions and purified as described previously [12,19]. Plasmid containing the coding sequence for full-length STAR-2 (Wormbase gene sequence #T21G5.5) was provided by Elizabeth Goodwin's laboratory (University of Wisconsin). The region corresponding to the STAR-2 STAR domain (residues 55-265) was amplified using PCR and inserted into pMal-c2x (NEB) plasmid for overexpression of STAR-2-STAR with an N-terminal MBP fusion. BL21(DE3) gold *E. coli *cells transformed with pMal-c2x-STAR-2 plasmid were grown in LB media to an OD_600 _of 0.6 whereby STAR-2 overexpression was induced by addition of 1 mM IPTG for 3 hours. Harvested cell pellets were resuspended and lysed by sonication followed by initial purification on an amylose resin (NEB) column. Pooled fractions containing STAR-2 were further purified by ion-exchange chromatography, first on a HiTrap Q column (Amersham Biosciences) followed by a HiTrap SP column. Pure STAR-2 protein was dialyzed against 4 L of storage buffer (20 mM Tris, pH 7.5, 20 mM NaCl, 2 mM DTT) and stored at 4°C for use in binding experiments.

### RNA preparation

All RNA constructs were chemically synthesized (Dharmacon). Lyophilized RNA pellets were resuspended in deprotection buffer and incubated at 60°C for 30 minutes. Deprotected RNAs were thoroughly dried in a SpeedVac, resuspended in deionized water and stored according to the manufacturer's protocol. 5'-Fluorescein-labeled RNA constructs used in Fp binding experiments were treated as above except all steps were conducted in the dark. RNAs used in gel mobility shift experiments were 5' radiolabeled by incubation with γ-^32^P-ATP (Perkin Elmer) and T4 polynucleotide kinase (NEB) for 1 hour at 37°C.

### Fluorescence-polarization (Fp) and Electrophoretic Gel Mobility Shift (EMSA) RNA binding assays

Complex formation between the various STAR proteins and RNA was monitored by both fluorescence-polarization and EMSA. For direct titration experiments, individual STAR proteins at a range of concentrations were titrated into a constant concentration of labeled RNA (1 nM fluorescein-labeled RNA for Fp or 100-300 pM radiolabeled RNA for EMSA) in reaction buffer (10 mM Tris, pH 8.0, 25 mM NaCl, 0.1 mM EDTA, 0.1 mg/mL tRNA, 5 μg/mL heparin and 0.01% IGEPAL CA630) and the reactions equilibrated at room temperature for 3 hours. Prior to equilibration with protein, labeled RNA was heated at 65°C for 2 minutes and allowed to cool to room temperature to remove any residual secondary structure.

Fp samples were equilibrated in a total volume of 100 μL in 96 well plates (Grenier) and every plate successively measured 10 times in a Packard Fusion plate reader to obtain the average polarization and standard deviation values for each protein concentration. Samples for gel mobility shifts were equilibrated in 20 μL total volume. 5 μL of each sample was loaded on a pre-run 6% native gel (29:1 acrylamide/bis-acrylamide, 0.5 × TBE) after addition of loading dye (30% v/v glycerol, bromophenol blue, xylene cyanol) and the gel run at 600 V for 30 minutes at 4°C. Gels were dried and exposed to a phosphorimager screen overnight. ImageQuant software (Molecular Dynamics) was used to quantify the fraction of labeled RNA in complex with protein. K_d _values for each RNA were calculated by fitting the binding data to a modified version of the Hill equation as described previously [19,25].

Competition Fp experiments employed the identical conditions as the direct titration experiments described above except that various concentrations of competitor RNA were titrated into a reaction mixture where a constant concentration of STAR protein had been added. Protein concentrations were chosen such that 70-90% of the labeled RNA would be in a bound complex. Polarization values were measured after plates had incubated at room temperature for 3 hours. IC_50 _values for each competitor RNA were determined by a fit of the data to the following equation using IGOR (Wavemetrics):

where f equals fraction bound or polarization value, C is the concentration of unlabeled competitor RNA, m is the maximum signal and b is the base signal. Apparent binding dissociation constants for competitor RNAs (K_i_^app^) were fit to a solved quadratic expression of the equation originally described by Lin and Riggs [26,27].

R, P, C, K_d _and K_c _are the concentrations of fluorescent RNA, protein, competitor RNA, and the dissociation constants for the wild type and competitor RNAs, respectively.

## Authors' contributions

AC, JW and KLB performed the experiments and analyzed the data. AC, KLB and JRW conceived the experiments and AC wrote the manuscript. All authors read and approved the final manuscript.
